# Viral co-infections among SARS-CoV-2-infected children and infected adult household contacts

**DOI:** 10.1007/s00431-021-03947-x

**Published:** 2021-01-27

**Authors:** Fiona Pigny, Noémie Wagner, Marie Rohr, Aline Mamin, Pascal Cherpillod, Klara M. Posfay-Barbe, Laurent Kaiser, Isabella Eckerle, Arnaud G. L’Huillier, for the Geneva Pediatric COVID Group, Alain Gervaix, Laurence Lacroix, Kevin Haddad, Klara M. Posfay-Barbe, Noemie Wagner, Arnaud G. L’Huillier, Selina Pinosch, Serge Grazioli, Riccardo Pfister, Barbara Widlhaber, Maurice Beghetti, Albane Maggio

**Affiliations:** 1 Laboratory of Virology, Geneva University Hospitals and Faculty of Medicine, 1211 Geneva, Switzerland; 2 Pediatric Infectious Diseases Unit, Geneva University Hospitals and Faculty of Medicine, 1211 Geneva, Switzerland; 3 Laboratory of Virology, Centre for Emerging Viral Diseases & Division of Infectious Diseases, Geneva University Hospitals and Faculty of Medicine, 1211 Geneva, Switzerland; 4 Laboratory of Virology & Centre for Emerging Viral Diseases, Geneva University Hospitals and Faculty of Medicine, 1211 Geneva, Switzerland

**Keywords:** COVID-19, SARS-CoV-2, Respiratory virus, Familial clusters, Co-infection

## Abstract

We evaluated the rates of viral respiratory co-infections among SARS-CoV-2-infected children. Twelve percent of SARS-CoV-2-infected children had viral co-infection with one or more common respiratory viruses. This was significantly more frequent than among their SARS-CoV-2-infected adult household contacts (0%; *p*=0.028). Compared to the same period the previous year, common respiratory viruses were less frequently detected (12% vs 73%, *p*<0.001).

*Conclusion*: Despite partial lockdown with school and daycare closure, and consequently similar exposure to common viruses between children and adults, SARS-CoV-2-infected children had more frequent viral respiratory co-infections than their SARS-CoV-2-infected adult household contacts. Circulation of common respiratory viruses was less frequent during the SARS-CoV-2 outbreak when compared to the same period last year, showing the impact of partial lockdown on the circulation of common viruses.

**Table:** 

**What is Known:** *• Viral respiratory co-infections are frequent in children.* *• SARS-CoV-2 can be identified alongside other respiratory viruses, but data comparing children and adults are lacking.*	
**What is New:** *• Children infected with SARS-CoV-2 are more likely to have viral respiratory co-infections than their SARS-CoV-2-infected adult household contacts, which is surprising in the context of partial lockdown with schools and daycare closed.* *• When compared to data collected during the same period last year, our study also showed that partial lockdown reduced circulation of common respiratory viruses.*

**What is Known:**

*• Viral respiratory co-infections are frequent in children.*

*• SARS-CoV-2 can be identified alongside other respiratory viruses, but data comparing children and adults are lacking.*

**What is New:**

*• Children infected with SARS-CoV-2 are more likely to have viral respiratory co-infections than their SARS-CoV-2-infected adult household contacts, which is surprising in the context of partial lockdown with schools and daycare closed.*

*• When compared to data collected during the same period last year, our study also showed that partial lockdown reduced circulation of common respiratory viruses.*

## Introduction

Viral respiratory infections are among the most common infections in adults and children. Molecular diagnostic testing allows to detect multiple concomitant respiratory viruses in the same clinical specimen. Respiratory viral co-infections are more frequent in symptomatic young children [1, 2]. Data from the pre-pandemic era are inconsistent about whether viral respiratory co-infections are associated with increased disease severity when compared to infections with a single virus but a meta-analysis suggested no significant difference [3]. Indeed, the mechanistic interactions between two respiratory viruses have not been clearly characterized: some viruses might potentiate or inhibit each other [2, 3]. The prevalence of viral co-infections varies between viruses, with picornaviruses or adenoviruses more likely to be identified during co-infections whereas influenza is more likely to be identified as a single pathogen [2]. Viral co-infections in SARS-CoV-2-infected adults range from 3 to 21% [4, 5], while they range between 5 and 19% in SARS-CoV-2-infected children [6]. The aim of our study was to evaluate rates of viral respiratory co-infections among SARS-CoV-2-infected children and compare it to SARS-CoV-2-infected adults living in the same households.

## Material and methods

Diagnostic testing was performed at Geneva University Hospitals’ laboratory of virology, a tertiary center that serves the Geneva region and also serves as the Swiss National Reference Centre for Emerging Viral Diseases. Symptomatic patients meeting local testing criteria and with a SARS-CoV-2-positive reverse-transcription polymerase chain reaction (RT-PCR) were identified through Geneva University Hospitals’ laboratory surveillance network, as previously described [7]. During the study period, every patient with respiratory symptoms or fever met testing criteria, independently of epidemiological links. There was no restriction in pediatric testing policies when compared to adults and no significant shortage of testing capacity in our institution during this time period. Children were hospitalized only in case of clinical indication, as per emergency room staff’s assessment.

RT-PCR-positive patients <16 years old were approached by the study team and household history of SARS-CoV-2 infection as well household RT-PCR results were obtained after informed consent. Household contacts were defined as persons living in the same housing as the SARS-CoV-2-infected child. The study was approved by the local ethics committee (protocol #2020-0835).

The NPS specimens were collected with a flocked swab in universal transport medium (Floqswab, Copan, Italy) and SARS-CoV-2 RT-PCR testing was performed as previously described [8]. Specimens were tested according to manufacturers’ instructions on various platforms over the course of the outbreak, to fulfill the increasing diagnostic demand such as in house methods using eMAG extraction (bioMérieux, France) and Charité RT-PCR protocols [9], BD SARS-CoV-2 reagent kit for BD Max system (Becton, Dickinson and Co, USA) and Cobas 6800 SARS CoV2 RT-PCR (Roche, Switzerland). The SARS-CoV-2-positive specimens were also tested for human metapneumovirus (hMPV), picornavirus, parainfluenza (PIV) 1/2/3/4, adenovirus (ADV), human coronavirus (HCoV) HKU1/229E/NL63/OC43, bocavirus, respiratory syncytial virus (RSV), and influenza A and B using either an in-house RT-PCR panel or the multiplex RT-PCR Fast-Track Diagnostics Resp21 commercial panel (Fast-Track Diagnostics, Esch-sur-Alzette, Luxembourg).

Groups were compared using chi-square or Mann-Whitney *U* tests depending on variable distribution. *P* values <0.05 were considered significant. Statistics were performed using SPSS version 23.0 (IBM SPSS Statistics, IBM Corporation).

## Results

Fifty-one children <16 years old with a positive SARS-CoV-2 RT-PCR were included in the analysis and had subsequent testing for common respiratory viruses. Median age was 11.0 years (interquartile range [IQR] 5.0–14.4) and 53% (27/51) were female. The most frequent diagnosis was upper respiratory tract infection (*n*=34), followed by influenza-like illness (*n*=4), obstructive bronchitis, community-acquired pneumonia (CAP) and fever without a source (FWS; *n*=2 each), sepsis-like syndrome, febrile gastroenteritis, croup, pharyngitis, and apparent life-threatening event (ALTE; *n*=1 each). For two patients who consulted outside our institution, the diagnostic was not clear because the chart was not available.

At least one additional respiratory virus was identified in 11.7% of cases (6/51). The detected viruses were picornaviruses (*n*=3), HCoV-NL63 (*n*=2), ADV (*n*=1), and hMPV (*n*=1). One of the children was co-infected with SARS-CoV-2, picornavirus, and hMPV. Neither influenza nor RSV were identified. Median age was lower among SARS-CoV-2-positive children presenting with a co-infection (4.2 years [IQR 1.7–7.3]) than without co-infection (12.3 years [IQR 7.1–14.5]; *p*=0.023). All patients with co-infection were managed in the ambulatory setting, whereas 6 of the 45 without co-infections were admitted to the general pediatrics ward (*p*=0.452). Indications for admissions were CAP (*n*=2), FWS (*n*=2), sepsis-like syndrome (*n*=1), and ALTE (*n*=1). Reported comorbidities of admitted children were hepato-renal polykistosis (*n*=1), asthma (*n*=1), and overweight (*n*=1). All children were discharged after 1–4 days without sequelae. Cycle threshold (CT) values of co-infecting viruses ranged between 23.8 and 33.1.

As a comparison, distribution of respiratory viruses was recovered from pediatric specimens (*n*=37) tested in our institution during the same period one year earlier (March 1 to April 30, 2019). The overall percentage of positive virus detection, with at least one respiratory virus identified, was 73.0% (27/37) in patients <16 years (*p*<0.001), with four patients co-infected with more than one respiratory virus. Here, the most frequently identified viruses were picornaviruses (*n*=14), hMPV (*n*=6), RSV (*n*=4), ADV and PIV3 (*n*=3 each), HCoV-NL63, HCoV-HKU1, and bocavirus (*n*=1 each). The median age in the 2019 cohort was however significantly younger than in 2020 (1.95 years [IQR 0.5–5.3] vs 11.0 years [IQR 5.0–14.4]; *p*<0.001).

We then compared viral co-infections between SARS-CoV-2-infected children and SARS-CoV-2-infected adults living in the same household. Twenty-eight household clusters among which at least an adult and a child tested positive for SARS-CoV-2 by RT-PCR were included, totalling 30 infected children and 41 infected adults (21 mothers, 15 fathers, 4 grand-parents, and 1 adult sibling). Median age of children was 9.3 years (IQR 3.4–14.0) and median age of adults was 43.0 years (IQR 36.5–52.0). Viral co-infection was identified in 13.3% of children (4/30) versus 0% of SARS-C0V-2 infected adults living in the same household (0/41; *p*=0.028). The identified co-infections were picornaviruses (*n*=3), hMPV (*n*=1), and HCoV-NL63 (*n*=1). Of note, two co-infections occurred in SARS-CoV-2-infected siblings but detected viruses were different (picornavirus and HCoV-NL63) and their mother only tested positive for SARS-CoV-2.

## Discussion

Our data show that during the peak of the SARS-CoV-2 outbreak in a severely affected area [10], viral respiratory co-infections were identified in 12% of the children with SARS-CoV-2 infection, which is similar to other reports of co-infections among children with COVID-19 [6, 11]. The most frequently identified viruses were picornaviruses, which are the most common respiratory pathogens in children [1]. In our cohort, children with co-infections were younger than those without, which is in line with previous data [11]. Both younger age and viral co-infections have been associated with more severe SARS-CoV-2 infection in children [11] but it is unclear whether both variables are independent risk factors of severe pediatric SARS-CoV-2 infection. In our dataset, the six SARS-CoV-2-infected children with co-infection were managed in the ambulatory setting, whereas 13% of the children without co-infection had to be admitted. Our data therefore do not suggest an increased disease severity in case of viral co-infection among SARS-CoV-2-infected children, at least in this small cohort of patients.

Respiratory viruses detected in our cohort of SARS-CoV-2-infected children were largely comparable to what was seen in the pre-pandemic period, with picornaviruses being the most frequently detected co-infecting virus. However, comparisons of circulating respiratory viruses between pre-pandemic and pandemic time periods need to be interpreted cautiously as they reflect different epidemiological situations.

The other important finding of our study was the significantly more frequent rate of viral co-infection among SARS-CoV-2-infected children when compared to adults living in the same household. The fact that viral respiratory co-infections are more frequent in children than adults has already been shown in the pre-pandemic era [2] and in one report of an early cluster of SARS-CoV-2 infections [12], before lockdown measures were in place, with only one child showing co-infection with SARS-CoV-2, picornavirus, and influenza A(H1N1) at the same time. Surprisingly, despite partial lockdown with schools and daycare closed [7], SARS-CoV-2-infected children were co-infected with other respiratory viruses whereas their parents were not. Because of the partial lockdown, one could have expected that the exposure to community-acquired viruses would be similar between adults and children living in the same household. One can wonder how these children acquired other viruses if they were confined at home with their parents. The identified co-infections might also reflect asymptomatic or post-symptomatic carriage with prolonged shedding, which is well frequent for these viruses [1], even though CT values were suggestive of a recently acquired infection. The co-circulation of SARS-CoV-2 with HCoVs, such as HCoV-NL63 identified in two patients, is not surprising and has been previously reported [4, 5].

There are a few limitations of the study. First, specimens used for the study were routine specimens and because of limited volume, some samples had to be diluted to assess for the presence of other respiratory viruses, potentially reducing the sensitivity. Second, based on the epidemiological context, individual indications to perform an NPS might have differed between 2019 and 2020, potentially contributing to explain differences in co-infection rates. Third, the transversal study design with a single NPS does not allow to characterize the sequence of viral co-infections and whether we detected asymptomatic or post-symptomatic SARS-CoV-2 carriage. Then, the sample size was limited, even though we were able to identify significant differences in co-infection rates between adults and children. Finally, while this has been previously published in large adult datasets [4, 5], having a control group of children and adults without SARS-CoV-2 infection would have provided interesting insights in viral respiratory infection rates among SARS-CoV-2-negative patients but because of limited storage capacity during the outbreak, SARS-CoV-2-negative specimens were discarded in our laboratory.

In conclusion, this study assesses viral respiratory co-infections in a relatively large dataset of SARS-CoV-2-infected children. Our data also show that despite partial lockdown, co-infection was significantly more frequent in SARS-CoV-2-infected children when compared to SARS-CoV-2-infected adults in the same households, despite an expected identical exposure to community-acquired viruses. Larger datasets are needed to confirm our findings and also to clarify whether both young age and viral co-infection are independent risk factors for severe pediatric COVID-19.

## Abbreviations

ADV: Adenovirus
CT: Cycle threshold
hCoV: Human coronavirus
hMPV: Human metapneumovirus
IQR: Interquartile range
PIV: Parainfluenza virus
RSV: Respiratory syncytial virus
RT-PCR: Reverse-transcription polymerase chain reaction
